# Ultralow-threshold multiphoton-pumped lasing from colloidal nanoplatelets in solution

**DOI:** 10.1038/ncomms9513

**Published:** 2015-09-30

**Authors:** Mingjie Li, Min Zhi, Hai Zhu, Wen-Ya Wu, Qing-Hua Xu, Mark Hyunpong Jhon, Yinthai Chan

**Affiliations:** 1 Department of Chemistry, National University of Singapore, 3 Science Drive 3, Singapore 117543, Singapore; 2 Institute of High Performance Computing A*STAR, 1 Fusionopolis Way, #16-16 Connexis, Singapore 138632, Singapore; 3 Institute of Materials Research & Engineering A*STAR, 3 Research Link, Singapore 117602, Singapore; 4 Microfluidics Systems Biology Lab, Institute of Molecular and Cell Biology A*STAR, 61 Biopolis Drive, Singapore 138673, Singapore

## Abstract

Although multiphoton-pumped lasing from a solution of chromophores is important in the emerging fields of nonlinear optofluidics and bio-photonics, conventionally used organic dyes are often rendered unsuitable because of relatively small multiphoton absorption cross-sections and low photostability. Here, we demonstrate highly photostable, ultralow-threshold multiphoton-pumped biexcitonic lasing from a solution of colloidal CdSe/CdS nanoplatelets within a cuvette-based Fabry–Pérot optical resonator. We find that colloidal nanoplatelets surprisingly exhibit an optimal lateral size that minimizes lasing threshold. These nanoplatelets possess very large gain cross-sections of 7.3 × 10^−14^ cm^2^ and ultralow lasing thresholds of 1.2 and 4.3 mJ cm^−2^ under two-photon (*λ*_exc_=800 nm) and three-photon (*λ*_exc_=1.3 μm) excitation, respectively. The highly polarized emission from the nanoplatelet laser shows no significant photodegradation over 10^7^ laser shots. These findings constitute a more comprehensive understanding of the utility of colloidal semiconductor nanoparticles as the gain medium in high-performance frequency-upconversion liquid lasers.

## Abstract (web summary)

Multiphoton-pumped lasing from semiconductor nanocrystals in solution is difficult due to Auger recombination, low volume fraction and high threshold. Here, Li *et al*. demonstrate photostable, ultralow threshold multi-photon pumped lasing from colloidal CdSe/CdS nanoplatelets in a Fabry-Pérot optical resonator.

## Graphical abstract

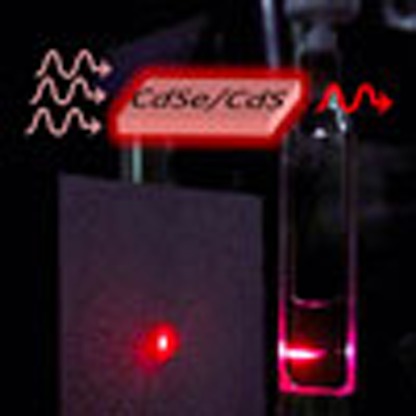

Colloidal semiconductor nanocrystals (NCs) are very attractive as optical gain media owing to their ease of fabrication, wide size-dependent colour tunability and spectrally narrow gain profiles^1^,^2^,^3^,^4^,^5^. Despite the first observation of optically pumped stimulated emission (SE) in a close-packed film of semiconductor NCs more than a decade ago^1^, only a very limited number of studies have documented the achievement of SE or lasing from NCs in solution so far^6^,^7^,^8^. Nevertheless, a solution-based gain medium can be highly desirable because of its flexibility of incorporation into optical cavities of any size and shape. For example, such a gain medium could be incorporated into optofluidic lasers^9^,^10^,^11^, which integrate microfluidics, a liquid gain medium and optical resonators within microchannels and which have already been utilized for sensitive bio/chemical intracavity detection^12^,^13^ and on-chip imaging^14^. Concurrently, significant efforts have been made in designing semiconductor NCs that can produce SE under excitation with a photon energy that is much lower than their bandgap through a multiphoton absorption process^15^,^16^,^17^,^18^. Emission from semiconductor NCs in solution obtained via multiphoton excitation can offer distinct advantages in the fields of nonlinear photonics and imaging *in vivo* that include deeper tissue penetration as well as less Rayleigh scattering, photodamage and photobleaching^19^,^20^,^21^,^22^. If these multiphoton-pumped (MPP) semiconductor NCs can be made to produce SE rather than spontaneous emission, they can be harnessed in the emerging field of optofluidic biolasers and can potentially improve the sensitivity and resolution of bio-imaging at the molecular, cellular and tissue level^23^,^24^,^25^.

Although MPP lasing has previously been realized using solutions of certain organic dyes^26^,^27^, semiconductor NCs can offer considerable advantages in terms of their relatively large multiphoton absorption cross-sections and high photostability^28^. However, to the best of our knowledge, neither MPP-amplified spontaneous emission (ASE) nor lasing has been observed from semiconductor NCs in solution, and only recently has three-photon-pumped SE been achieved in a close-packed film of NCs^18^. One major impediment is the extremely fast non-radiative Auger recombination of multiexcitons in semiconductor NCs or nanorods that prevents sustained population inversion and therefore optical gain^1^. For the achievement of SE, the SE build-up time, which is reciprocally related to the volume fraction of NCs within the gain medium, should be faster than the Auger-dominated lifetime of the gain excited state. Understandably, it is very difficult to achieve lasing in solution given the low volume fraction of NCs and relatively small gain cross-section on a per NC basis (for example, ∼10^−17^ cm^2^ for 2.6 nm diameter CdSe NCs^1^). Furthermore, the threshold pump intensity for MPP SE is typically much higher than that for one-photon excitation^17^, making it challenging to prevent artifacts such as solvent boiling or evaporation within a practical device. Overcoming these obstacles requires semiconductor nanostructures with large multiphoton absorption cross-section and reduced multiexcitonic Auger recombination rates.

Recently, colloidal quasi-two-dimensional (2D) quantum wells, also known as semiconductor nanoplatelets (NPLs) have attracted much attention as a new class of solution-processable optical gain media since they retain many of the salient features of semiconductor NCs while also possessing high oscillator strengths, large absorption cross-sections and strongly suppressed Auger recombination rates^29^,^30^.

Herein, we demonstrate that two- and three-photon-pumped (2PP and 3PP) lasing from colloidal CdSe/CdS core/shell NPLs in solution can be achieved using a cuvette-based Fabry–Pérot cavity. Laser emission from the MPP NPL solution was highly photostable, thermally stable, directional and polarized. The concentration and lateral size of NPLs were found to have significant effects on the lasing threshold. The gain cross-section per NPL was determined to be a staggering ∼7.3 × 10^−14^ cm^2^, and the lasing threshold pump fluence (*F*_th_) in solution was as low as ∼1.2 and ∼4.3 mJ cm^−2^ for two- and three-photon excitation respectively. Remarkably, the lasing thresholds measured here are ∼3–10 times lower than those previously reported for MPP ASE from spherical semiconductor NCs in a closed-packed matrix despite the higher volume fraction^18^,^31^,^32^. These findings collectively highlight the potential of colloidal semiconductor NPLs as gain media for high-performance upconversion lasers, and provide guidelines for utilizing them in optofluidic light amplification and stimulated emission-based biophotonic techniques.

## Results

### Synthesis and characterization

**Figure 1:**
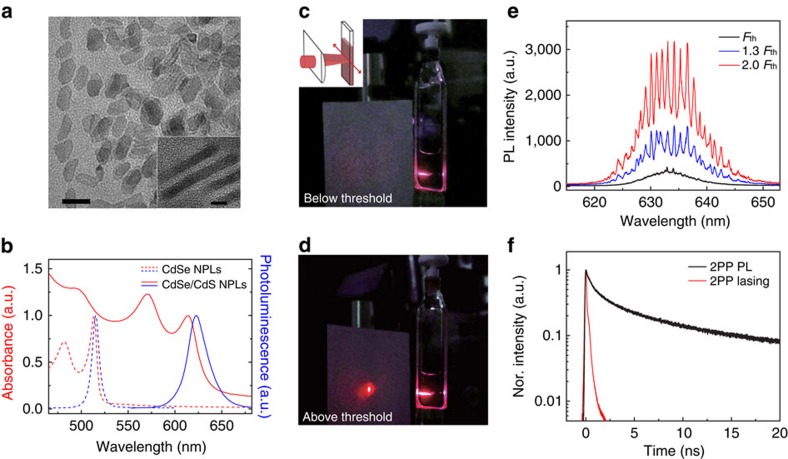
MPP lasing from NPLs solution in a cuvette cell. (**a**) TEM image of sample NPL390 (scale bar, 25 nm). Inset shows the HRTEM of standing NPLs (scale bar, 5 nm). (**b**) Absorption (red) and photoluminescence (blue) spectra of the CdSe (dash) and CdSe/CdS core/shell NPLs (solid). They are normalized at the first excitonic transitions. Photographs show the transversely excited cuvette filled with NPL solution under 800 nm femtosecond laser (1 kHz, 150 fs) excitation at the pump fluence (**c**) below (0.9*F*_th_) and (**d**) above lasing threshold (1.3*F*_th_). *F*_th_=1.2 mJ cm^−2^. The distance between the screen and cuvette is 3.5 cm. Inset shows a schematic experimental configuration. (**e**) Lasing spectra of sample NPL390 in solution collected at the edge of cuvette under different pump fluence. (**f**) Fluorescence decay profiles of spontaneous emission (black) and 2PP lasing (red).

Colloidal CdS/CdSe core/shell NPLs with different lateral dimensions used in this study were synthesized by growing two monolayers of CdS shells on either side of four monolayer thick CdSe NPLs using a wet-chemical atomic layer deposition technique^29^ as described in detail in the Supplementary Methods. Figure 1a shows representative transmission electron microscope (TEM) images of core/shell CdSe/CdS NPLs with a thickness of ∼2.75 nm, average edge dimensions of ∼15 × 25 nm and a resulting lateral area of ∼390 nm^2^ (named as NPL390). Overcoating the CdSe NPLs with two monolayers of CdS resulted in the band-edge absorption red shifting from 513 to 613 nm while the spontaneous emission was shifted from 515 to 622 nm (Fig. 1b). These findings are similar to previous observations of CdSe/CdS core/shell NPLs^29^.

### 2PP and 3PP lasing from NPLs solution

To generate MPP lasing in solution, as-synthesized NPLs were ligand exchanged with and dispersed in excess 5-amino-1-pentanol (AP) at a concentration of *C*_0_=21.6 μmol l^−1^ (μM) (see Supplementary Note 1 for the determination of concentration). The mixture of NPLs in AP served as the liquid gain medium in a quartz cuvette with a 1-cm-wide and 1-mm-thick optical pathlength (Fig. 1c). Our choice of AP as the ligand and solvent was motivated by its good affinity for the surface of the NPLs and the excellent dispersibility and colloidal stability conferred even at very high NPL concentrations. The cuvette was transversely excited by a pulsed laser source with a ∼150 fs pulse width and 1 kHz repetition rate. The excitation beam was focused into a stripe using a cylindrical lens, and the output emission was collected from one of the side faces of the cuvette in a direction approximately normal to the incident pump beam as schematically shown in the inset of Fig. 1c. Here, the two parallel optical windows of the cuvette can act as mirrors, thereby providing optical feedback for lasing in a manner akin to a planar Fabry-Pérot etalon cavity. The optical pump wavelengths were 800 and 1,300 nm for two-photon and three-photon excitation, respectively. For 800 nm excitation below threshold on sample NPL390, a striped beam of red light could be seen on the cuvette but not on a screen placed to its side (Fig. 1c). At excitation intensities above threshold, an intense spot of light could be observed on the screen as shown in Fig. 1d, thus signifying the onset of 2PP lasing (see Supplementary Fig. 1 for a photograph illustrating 3PP lasing). The near- and far-field intensity distribution of the 2PP and 3PP lasing at a pump fluence of 1.5*F*_th_ were also characterized, yielding divergence angles commensurate with previously reported cuvette-based lasers^27^ (Supplementary Fig. 2, Supplementary Note 2 and Supplementary Table 1).

Figure 1e shows the 2PP lasing spectra at different pump fluence above threshold, with the emission peak located at ∼634 nm. At larger pump intensities, well-resolved, equally spaced resonant modes were observed. A similar periodic pattern of lasing spectra was previously observed from a solution of Coumarin dye in a transversely excited cuvette^33^. Such spectral modulation was attributed to a Fabry–Pérot cavity formed by the front and back windows of the quartz cuvette. The wavelength spacing of two adjacent maxima is determined via the following relation^33^:


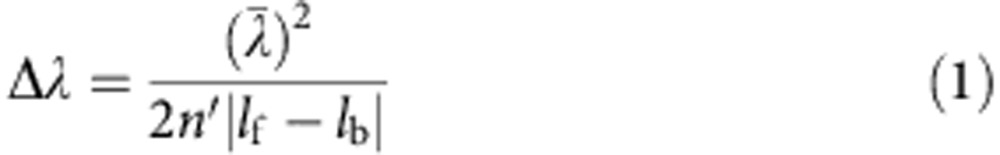


where 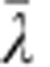 is the average lasing wavelength, *n*' is the refractive index of quartz, *l*_f_ and *l*_b_ are the thicknesses of the front and back windows of the cuvette. The value of |*l*_f_–*l*_b_| was found to be 115±5 μm by a digital micrometre. The calculated Δ*λ* of 1.1±0.1 nm agrees with the experimental spacing of ∼1.0 nm as seen in Fig. 1e, which further corroborates the fact that our cuvette functioned as a Fabry–Pérot cavity.

We also determined that the cuvette used in our experiments possessed a relatively high quality factor (*Q*) of ∼1,200 as calculated by *Q*=*λ*/δ*λ*. Here *λ* refers to the peak wavelength of the lasing transition while δ*λ* (∼0.54 nm) is its corresponding full-width at half-maximum. The width of the entire set of peaks is about 10 nm, and can likely be made narrower by reducing the size dispersity of the NPLs. In addition, the emission decay lifetime decreased sharply from ∼1.9 ns to a value approaching the instrument response time (∼50 ps) of our time-correlated single-photon counting setup below and above threshold (Fig. 1f). It should be noted that during the course of the measurements, evaporation of the solvent or microscopic aggregation of the NPLs were not observed. Taken together, these findings indicate strongly that 2PP lasing from NPLs in solution was successfully achieved.

### Effect of reabsorption on lasing

**Figure 2:**
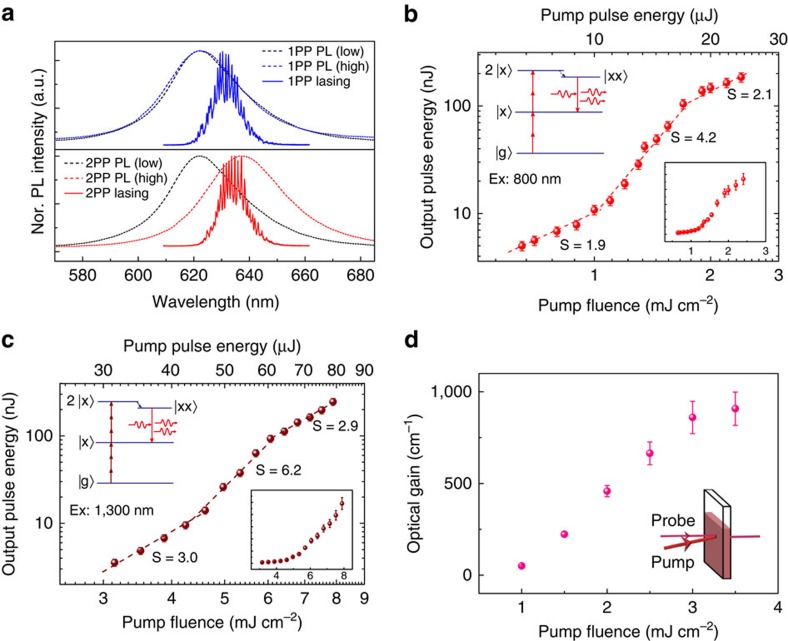
Biexcitonic lasing and optical gain. (**a**) Normalized (Nor.) 1PP and 2PP PL (dashed line) and lasing (solid line) spectra for sample NPL390 in solution. Low and high refer to the NPL concentration of ∼0.001*C*_0_ and *C*_0_, respectively. The concentration for 1PP and 2PP lasing is *C*_0_. The log-log plots of laser output energy versus pump fluence/energy together with the slopes (S) excited with (**b**) 800 nm and (**c**) 1,300 nm light for two- and three-photon excitation, respectively. Linear plots of laser output energy versus pump fluence are at the lower right insets of **b** and **c**. (**d**) Optical single-pass gain versus pump fluence for sample NPL390 in solution with concentration of *C*_0_. Inset shows the experimental configuration. The error bars were determined based on the standard deviation of four repeated acquisitions.

At the high NPL concentrations necessary to attain SE, it was found unexpectedly that the 2PP lasing peak was located at the higher energy side of its photoluminescence (PL) (Fig. 2a). In contrast, the one-photon pumped (1PP) lasing peak emerges from the lower energy side of its PL, as expected from a positive biexciton-binding energy 
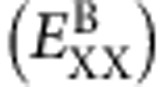
 (ref. 30). It is worth noting that due to a considerable overlap of the absorption tail with the PL (Fig. 1b), as well as a relatively large penetration depth of the excitation stripe into the sample under MPP conditions, reabsorption of emission under multiphoton excitation^34^ can be very significant for high concentrations of NPLs. This in turn causes a red shift in the spontaneous emission, which as shown in Fig. 2a, can be as large as ∼16 nm. Conversely, no obvious difference in PL spectra was observed from the low- and high-concentrated NPLs solution under one photon excitation because of its much shallower penetration depth. The 2PP lasing peak, however, has only a slight ∼3 nm red shift compared with the 1PP lasing peak because at the high excitation intensities required to achieve population inversion, the first excited state of most of the NPLs is filled and the corresponding absorption transition is bleached out. Therefore, the emergence of the 2PP lasing peak at the higher energy side of its 2PP PL spectrum is not due to the occurrence of a negative biexciton energy of interaction but rather the effect of reabsorption.

### Characteristics of MPP biexcitonic lasing

The output energy per pulse versus pump fluence from NPL solution laser is given in the log–log plot in Fig. 2b, where the s-shaped profile suggests a distinct transition from 2PP PL to 2PP lasing and then saturation. For comparison, a linear plot of the same data is provided in the lower right figure inset, where a clear threshold behaviour is readily observed. The quadratic power dependence obtained in the low excitation regime is attributed to spontaneous emission via two-photon absorption (2PA). The transition to a quartic power dependence of the output energy with pump fluence, which is accompanied by a narrowing of the emission spectrum into a few sharp peaks as illustrated in Fig. 2a (red curve), indicates the onset of lasing. The fourth power dependence is consistent with the assumption that the origin of the optical gain is largely biexcitonic in nature since the NPLs are excited from their ground to single exciton states and then subsequently to biexciton states (as shown schematically in the upper left inset of Fig. 2b). The power dependence returns to a quadratic slope at pump powers well above the lasing threshold, which is expected from gain saturation. 
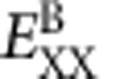
 was determined by the energy difference (∼30 meV) between the single-exciton emission peak at very low pump fluence and the fitted biexciton emission peak at high pump fluence of a 1PP solution of NPLs at low concentration (Supplementary Fig. 3 and Supplementary Note 3).

In the case of 3PP lasing, the output energy versus pump fluence plot shows a trend analogous to that of the 2PP experiment. Spontaneous emission at low excitation fluence yielded a cubic dependence of the output intensity on pump energy while the onset of lasing at higher fluence was accompanied by a sixth power dependence, as shown in Fig. 2c. As with the 2PP data, the fitted exponents to the output intensity under 3PP agrees with a biexciton gain model, as illustrated in the upper left inset of Fig. 2c. At large pump intensities, the relationship between output and input energy is again approximately cubic, signifying gain saturation. A corresponding linear plot is given in the lower right inset of Fig. 2c to aid in visualizing clear threshold behaviour and the onset of optical amplification.

For sample NPL390 at a concentration of *C*_0_=21.6 μM in AP, *F*_th_ for 2PP lasing was as low as ∼1.2 mJ cm^−2^, which is about 5–10 times smaller than 2PP ASE from close-packed films of CdZnS/ZnS NCs^31^, and CdSe/CdS/ZnS NCs^32^. Similarly, the threshold for 3PP lasing was determined to be ∼4.3 mJ cm^−2^, which is about ∼3 times lower than that for 3PP ASE in close-packed films of CdSe/CdS/ZnS NCs^18^. The average number of initially generated excitons per NPL (<N>) can be calculated by using <N>_2PP_=*F*^2^*σ*_2_*τ*^−1^ and <N>_3PP_=*F*^3^*σ*_3_*τ*^−2^, where *F* is the pump fluence, *τ* is the pulse duration, and *σ*_2_ and *σ*_3_ are the two-photon and three-photon absorption cross-sections measured by Z-scan technique^35^,^36^, respectively. The Z-scan curves (Supplementary Fig. 4) and detailed data processing for the extraction of *σ*_2_ and *σ*_3_ are given in the Supplementary Note 4. For the sample NPL390, *σ*_2_ and *σ*_3_ are about 7.9 × 10^−45^ cm^4^ s per photon and 1.5 × 10^−75^ cm^6^ s^2^ per photon^2^, these values are 2–4 orders of magnitude larger than that of previously reported semiconductor NCs and about 4 orders of magnitude larger than that of typical organic dyes^34^,^37^,^38^. On the basis of the threshold pump fluence, the values of <N>_2PP-th_ and <N>_3PP-th_ are calculated to be ∼1.2 and ∼1.5 for 2PP and 3PP lasing, respectively, which is consistent with what is known of NCs exhibiting biexcitonic gain^1^.

To estimate the energy conversion efficiency of our MPP cuvette-based NPL laser, the ratio of the output energy to the absorbed excitation energy was determined. As shown in Fig. 2b, at a pump energy level of 20 μJ, the output energy per pulse of 2PP lasing is ∼150 nJ. At this pump level, the attenuation ratio of the pump energy due to 2PA was measured to be 0.42. Assuming that laser emission from both ends of the symmetric excitation stripe are identical, the overall energy conversion efficiency in the absence of absorption losses by unexcited regions of the gain solution (as observed in Fig. 1d) will be *η*_2pp-out_=3.6%. Similarly, for 3PP lasing, *η*_3pp-out_=1.2%. Although efficiencies as large as ∼10% in cuvette-based dye lasers have recently been reported^27^, further improvements to the quantum yield or absorption cross-section of the NPLs should allow for a substantial increase in their lasing energy conversion efficiency.

### Gain cross-section per NPL

The optical gain of a solution of NPLs, which provides some indication of lasing performance, was determined by pump–probe transmission measurements. Figure 2d shows the measured single-pass gain *g* for the sample NPL390 at different pump fluence under two-photon excitation (see Supplementary Note 5 for the details of the determination of *g* from pump–probe measurements). It is seen that *g* increases gradually and then reaches a maximum value *g*_max_ of 950±100 cm^−1^ at ∼3 mJ cm^−2^ as a result of gain saturation. Under this high pump fluence, virtually all photoexcited NPLs undergo complete population inversion and the NPL solution reaches its maximum gain, which we denote as *g*_max_. The gain cross-section per NPL (*γ*_*g*_) can be obtained via the relation *γ*_*g*_≈*g*_max_/*C*_0_ and was found to be 7.3 × 10^−14^ cm^2^. For comparison, this value of *γ*_*g*_ is about three orders of magnitude greater than that of one-photon excited 2.6 nm diameter CdSe (ref. 1) and 8.5 nm diameter CdZnS/ZnS NCs^8^. Notably, the gain cross-section per unit volume (that is, ∼6.7 × 10^5^ cm^−1^) is at least two orders of magnitude greater than these NCs, which may be attributed to the higher density of states available for SE in NPLs^29^.

### Minimum concentration of NPLs for lasing

The concentration of NCs in solution is a critical factor for determining the threshold pump fluence to achieve lasing. With the above obtained value of *γ*_*g*_, the minimum concentration *C*_min_ required to achieve lasing can be estimated. It is well known that threshold gain (*g*_th_) should be equal to the sum of the optical losses of the cavity. For our cuvette-based Fabry–Pérot cavity, we have the following relation^39^:


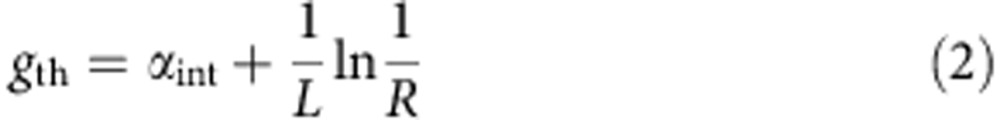


where *L* (=0.1 cm) is the cavity length (that is, the optical pathlength of the cuvette in Fig. 1c), *R* (∼0.04) is the reflectivity between air and quartz. *α*_int_ is the internal loss coefficient per unit length primarily due to free carrier absorption of NPLs. The second term on the right-hand side of equation (2) represents the mirror loss which was calculated to be ∼32 cm^−1^. At the minimum concentration of NPLs required for lasing, the threshold gain should be equal to its maximum gain, therefore *g*_th_=*γ*_*g*_*C*_min_. *α*_int_ can be neglected due to near-complete absorption bleaching of the band-edge under high pump fluence. Under these conditions, equation (2) reduces to 950*C*_min_/*C*_0_=32, which yields a minimum concentration *C*_min_≈0.03*C*_0_.

**Figure 3:**
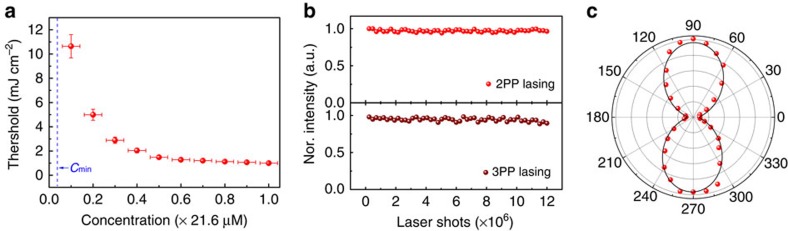
Concentration effect, photostability and polarization of laser. (**a**) Threshold pump fluence of 2PP lasing for sample NPL390 in solution at different concentrations. The horizontal error bars were determined based on the uncertainty associated with obtaining the molar extinction coefficient of the NPLs and the vertical error bars were determined based on the standard deviation of four repeated acquisitions. (**b**) The normalized (Nor.) peak intensity of 2PP and 3PP lasing as a function of laser shots. (**c**) The laser emission above threshold as a function of angle *θ* between the plane of the polarizer in front of detector and a plane orthogonal to the cuvette surface. The solid black line is the fitting based on sin^2^ *θ*.

The threshold pump fluence of 2PP lasing for different concentrations of sample NPL390 were also measured, and the results are plotted in Fig. 3a. It is readily observed that *F*_th_ initially increases slowly as the concentration is reduced and then increases rapidly at concentrations below ∼0.4*C*_0_. The lowest experimentally determined NPL concentration capable of achieving lasing was ∼0.1*C*_0_ (that is, 2.16 μM) with a corresponding pump threshold of ∼10.6 mJ cm^−2^, which is in reasonable agreement with *C*_min_. It is possible that the experimental minimum concentration may be lower, however our use of AP as the solvent resulted in boiling at the higher pump intensities required to achieve lasing in sample concentrations lower than 0.1*C*_0_.

### Photostability and polarization

Figure 3b illustrates the photostability of a MPP semiconductor NPL solution-based laser under continuous excitation with the 1 kHz femtosecond pump source. No noticeable degradation in the 2PP and 3PP laser emission intensities was observed over ∼1 × 10^7^ laser pulses (corresponding to ∼3 h of excitation) at a pump fluence approximately twice higher than that of the lasing threshold. While longer excitation periods were not explored, it should be mentioned that the number of laser pulses the solution sample was exposed to without significant degradation observed was already two orders of magnitude larger than the ASE from solutions of organic dyes^40^, and at least one order of magnitude larger than that from 3PP close-packed CdSe/CdS/ZnS films^18^. The excellent photostability afforded by CdSe/CdS NPLs in AP, and the absence of solvent evaporation within the pump intensity employed, provide further evidence that our choice of gain material and solvent provides a promising route to a robust, high-performance liquid gain medium. In addition, it is often advantageous for many applications if the laser output is linearly polarized. Figure 3c shows that the lasing above threshold from 2PP NPLs in the cuvette was strongly polarized normal to the plane of incidence (TE polarization) to an extent of about 90%, which is comparable to dye lasers^41^. Notably, thermal stability measurements from 23–120 °C showed little variation in terms of threshold or polarization (Supplementary Fig. 5 and Supplementary Note 6).

### Optimum NPL size for lowest threshold

**Figure 4:**
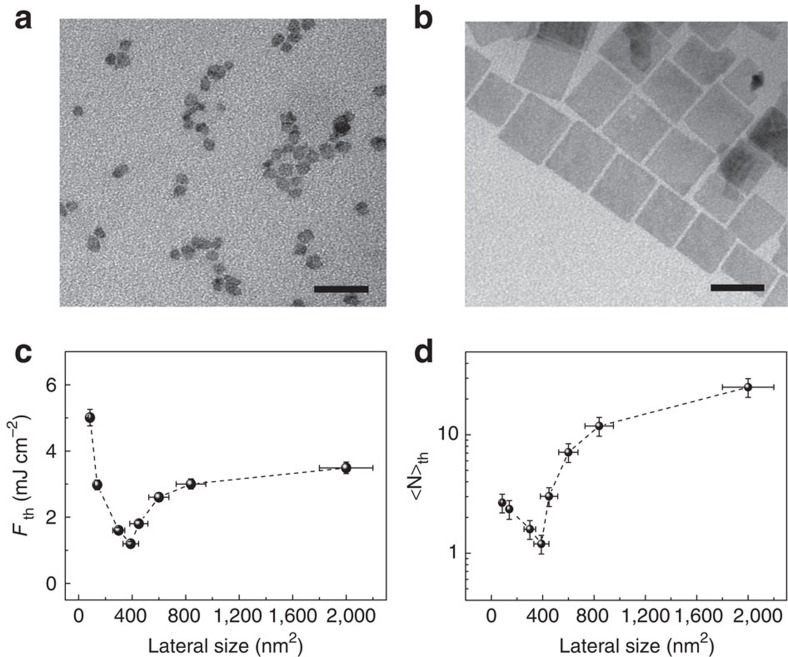
Effect of lateral size on lasing threshold. TEM images of NPLs with average lateral size of (**a**) 85 nm^2^, (**b**) 2,000 nm^2^. Both scale bars, 50 nm. (**c**) Threshold pump fluence and (**d**) initially generated of excitons per NPL at threshold pump fluence as a function of lateral sizes for 2PP lasing. The horizontal error bars were determined based on the lateral size distribution of the NPLs while the vertical error bars were determined based on the standard deviation of 4 repeated acquisitions.

In addition to the number density effects analysed previously, the lateral dimensions of NPLs can also play a critical role in determining their lasing threshold. To explore the size-dependent behaviour of NPLs for low-threshold lasing, eight batches of NPLs with varying lateral dimensions of ∼9 nm × 9.5 nm to ∼44 nm × 44 nm (corresponding to areas of ∼85 nm^2^ to 2,000 nm^2^) but with the same thickness (∼2.75 nm, see Supplementary Fig. 6 for HRTEM images) were investigated. Representative TEM images of the NPLs of small and large sizes are depicted in Fig. 4a,b. For the MPP lasing measurements, the concentrations of the different sized NPLs were adjusted to a constant value of *C*_0_=21.6 μM. It should be noted that the concentration of NPLs at relatively low concentration ranges can dramatically affect the threshold pump fluence whereas it is relatively insensitive to differentiate between high concentration values, as illustrated in Fig. 3a. Therefore, a high concentration of *C*_0_ was used for all samples so that uncertainties in the measurement of *F*_th_ as a result of errors in determining NPL concentration were minimal.

A clear trend was observed for the threshold pump fluence *F*_th_ as a function of the lateral size of the NPLs for both 2PP (Fig. 4c) and 3PP lasing (Supplementary Fig. 7). In both cases, *F*_th_ rapidly declines as the lateral size of the NPL increases, reaches a minimum, increases again and plateaus at the largest sizes measured. To gain deeper insight into the dependence of pump threshold on the lateral size of the NPLs, we determined the average number of excitons per NPL (<N>_th_) at the onset of 2PP lasing. This is an alternate measure for threshold that accounts for differences in the two-photon absorption cross-section for the various-sized NPLs. As shown in Fig. 4d, the dependence of <N>_th_ on the lateral size of the NPLs mirrors the behaviour of the pump threshold: <N>_th_ likewise exhibits a minimum at the same lateral size. This surprising threshold behaviour is not reconcilable with our general expectation that, for solutions at the same particle concentration but with different-sized NPLs, the larger-sized NPLs can achieve lower lasing threshold because of their large absorption cross-section (Table 1) and larger volume fractions. Further, the threshold behaviour cannot be attributed to variations in quantum yield because the deviation in quantum yield amongst the different-sized samples was small (<3.9%, see Table 1) and does not correlate with the trends observed in Fig. 4c,d.

We rationalize the minimum in the threshold by recognizing that quantum confinement can play a critical role in lasing. Given that the thicknesses of the different-sized NPLs were essentially the same, any differences in the extent of quantum confinement between different-sized NPLs should arise primarily from variations in their lateral dimensions. We estimate the radius of the biexciton to be ∼2.74 times of the 2D exciton Bohr radius^42^. Taking the 2D Bohr radius *α*_B_ in a CdSe NPL to be about 3.5 nm (ref. 43), we find that the biexciton radius should be about ∼9.6 nm. This is much smaller than the linear size of the largest particles and so we conclude that the large particles are in the weak confinement regime. As the lateral size of an NPL is decreased, its behaviour will gradually cross over into the strong biexciton confinement limit as the lateral size approaches the 2D biexciton size. Experimental evidence of lateral quantum confinement with decreasing NPL size can be observed from the slight blue shift in core CdSe NPL emission, which is accompanied by a larger red shift after overcoating with CdS (Supplementary Fig. 8) as was previously observed in small CdSe nanoparticles coated with a shell of ZnS (ref. 44).

The transition to strong confinement is generally accepted to be accompanied by a sharpening of the density of states and subsequent narrowing of the gain spectra^45^. This is a significant effect. A density matrix theory of lasers can be used to show that the peak gain can be increased by an order of magnitude at the same carrier density by transitioning from a quantum well structure to a quantum box structure^46^. The increase in gain at a given carrier density is reflected by a lowering of the pump threshold required for lasing. We also note that 
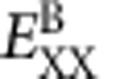
 of our NPLs in the intermediate confinement regime is fairly small (∼30 meV), on the order of *k*_B_*T*. This binding energy is expected to decrease with reducing the quantum confinement^46^. This means that 
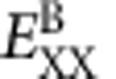
 for larger NPLs may drop below *k*_B_*T*, allowing for biexcitons to dissociate into uncorrelated excitons. In addition, the internal loss is expected to be higher for larger sized NPLs due an increase in free carrier absorption and scattering. These considerations collectively indicate that by reducing lateral size, the threshold number of excitons per NPL will be reduced, in agreement with our observations for NPLs with lateral areas of ∼400–2,000 nm^2^.

However, as the NPL size decreases further, the increased quantum confinement will inevitably lead to faster Auger recombination, which is known to progress at a rate inversely proportional to the volume of the particle for simple, spherical single component quantum dots^1^. Auger recombination rates will diverge and cause non-radiative recombination to eventually dominate carrier lifetime. We interpret the increasing *F*_th_ for very small NPLs (<400 nm^2^) to reflect the stronger pumping required to compensate for increased Auger recombination. The optimum lateral area of NPLs for lowest lasing threshold thus corresponds to a size where the competing size effects of density of states, 
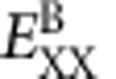
, internal loss and non-radiative Auger recombination are balanced.

## Discussion

In summary, low threshold two-photon (*λ*_exc_=800 nm) and three-photon (*λ*_exc_=1,300 nm) -pumped red lasing were successfully demonstrated in colloidal CdSe/CdS core/shell NPLs dispersed in 5-amino-1-pentanol within a cuvette-based optical cavity. The laser emission was found to be extremely photostable, enduring >10^7^ laser shots without photodegradation or solvent evaporation. This is advantageous to colloidal semiconductor NC-based solid state lasers where a fixed subpopulation of particles are constantly excited. Among eight batches of NPLs with average lateral sizes ranging from ∼85 to 2,000 nm^2^, NPLs with a lateral size of ∼390 nm^2^ (NPL390) which is close to the 2D biexciton size were found to exhibit the lowest threshold pump fluence and <N> required to achieve the lasing. For these NPLs, the lasing thresholds were extremely low (for example, ∼1.2 and 4.3 mJ cm^−2^ per pulse under two- and three-photon excitation for NPLs solution with the concentration of 21.6 μM, respectively) and was mainly attributed to an increased density of states that resulted from increased lateral quantum confinement. The maximum optical gain of the solution of sample NPL390 measured by pump–probe technique was relatively large at ∼950 cm^−1^. The magnitude of the deduced gain cross-section was determined to be ∼10^−14^ cm^2^, which is three orders of magnitude larger than those reported for colloidal NCs^1^,^8^. In addition, it was found that the threshold pump fluence increased slowly and then rapidly as the concentration of NPLs in the sample was reduced. Accordingly, a judicious choice of NPL size and concentration should yield the best performance to material consumption ratio.

Our findings provide guidelines for designing low threshold solution-based NPL lasers, and should also be extendable to their solid state counterparts. Photo- and thermally stable, multiphoton pumped solution-based colloidal NPL gain media are good candidates for frequency upconversion optofluidic lasers. This colloidal gain media opens new possibilities in nanolasing. For example, our system lends itself to integration into plasmonic waveguides that could allow for cavity-free lasing using the stopped-light concept^47^. Because the gain medium in a solution-based system can be changed on-the-fly, it is possible to engineer its properties independently of device-level properties such as optical confinement^48^. Overcoming the challenge of modifying the surface of NPLs to make them water dispersible and biocompatible while still preserving their desirable lasing properties will undoubtedly expand their utility as nonlinear optical biosensors.

## Methods

### Synthesis of CdSe/CdS nanoplatelets

Core/Shell CdSe/CdS NPLs with different lateral dimensions were synthesized according to previous method^29^ with slight modifications (see Supplementary Methods for details). Briefly, four monolayer thick CdSe core NPLs of various lateral sizes were overcoated with CdS by depositing alternating atomic layers of Cd and S until about two monolayers thick CdS shells were obtained. The as-synthesized core/shell CdSe/CdS NPLs were dissolved in *N*-methylformamide and then processed via two cycles of precipitation and re-dispersion in ethanol and *N*-methylformamide. The resulting particles were subsequently dried under nitrogen and then dispersed in 1-amino-5-pentanol to yield concentrated but aggregation-free solutions of colloidal NPLs.

### Optical characterization

PL quantum yield of NPLs in AP solution was measured using a Horiba Fluorolog-3 fluorescence spectrometer equipped with a Quanta- QUOTE integrating sphere. The excitation source for the lasing, time-resolved PL, pump-probe and Z-scan measurements was a femtosecond Ti-sapphire laser system (Spectra-Physics) with a repetition rate of 1 kHz and a pulse duration of ∼150 fs. The wavelength of the pump laser was tuned by an optical parametric amplifier (Light conversion, TOPAS). For the optical pumped lasing experiments, the laser pulses were focused by a cylindrical lens (with focal length=20 cm) into a stripe with a spot size of ∼5 mm × 0.2 mm (see Supplementary Fig. 9 for the pump beam profile) onto the surface of a cuvette whose optical pathlength was 1 mm. The direction of the pump laser was normal to the surface of the cuvette window. The emission was collected from one of the edges of the cuvette by an optical fibre coupled to a spectrometer (Horiba iH320) and detected by a CCD camera (Synapse, Horiba) or a power metre (Fieldmate, Coherent) with a high-sensitivity optical sensor (OP-2 Vis, Coherent) used for measuring emission energy. Emission lifetimes were measured using a time-correlated single-photon counting module (PicoQuant PicoHarp 300). For the Z-scan measurements, the cuvette containing the solution of NPLs was mounted on a linear delay stage and moved along a direction parallel to the propagation of the pump beam over the focal length of the lens (focal length=20 cm). Subsequently, the transmitted light was collected by a detector without any aperture placed in front of it. For pump–probe measurements, the probe beam was generated by focusing 800 nm wavelength laser pulses into a 1 mm sapphire plate in order to generate white-light continuum. The probe beam with a focused spot size of ∼20 μm in diameter was allowed to vertically pass through a solution of NPLs kept in a very thin cuvette (optical pathlength=200 μm) and the pump pulse with a focused spot size of ∼50 μm in diameter was overlapped with the probe pulse. The angle between the probe and pump beams was ∼30 degrees. The intensity of the transmitted probe pulse at the wavelength of the NPL sample lasing peak (∼634 nm) passing through a monochromator (MicroHR, Horiba) was measured by a lock-in-amplifier (SR830, Stanford Research Systems).

## Additional information

**How to cite this article:** Li, M. *et al*. Ultralow-threshold multiphoton-pumped lasing from colloidal nanoplatelets in solution. *Nat. Commun*. 6:8513 doi: 10.1038/ncomms9513 (2015).

## Supplementary Material

Supplementary Information Supplementary Figures 1-9, Supplementary Table 1, Supplementary Notes 1-6, Supplementary Methods and Supplementary References

## Figures and Tables

**Table 1:** Summary of quantum yield and 2PA and 3PA cross-sections of NPLs with different average lateral sizes.

Lateral size (nm^2^)	85	140	300	390	450	600	840	2,000
Quantum yield (%)	24	17	24	26	23	18	20	15
*σ*_2_ (× 10^−45^ cm^4^ s per photon)	1.3	2.8	6.0	7.9	7.4	8.9	12.5	19.8
*σ*_3_ (× 10^−75^ cm^6^ s^2^ per photon^2^)	0.5	0.7	1.2	1.5	1.8	3.0	3.9	4.4
