# Supplementary material for: Ultralow-threshold multiphoton-pumped lasing from colloidal nanoplatelets in solution
Source: Nat Commun. 2015 Sep 30;6:8513. doi: 10.1038/ncomms9513 (PMC4598837; doi:10.1038/ncomms9513)
Supplement: Supplementary Information — Supplementary Figures 1-9, Supplementary Table 1, Supplementary Notes 1-6, Supplementary Methods and Supplementary References [file ncomms9513-s1.pdf]

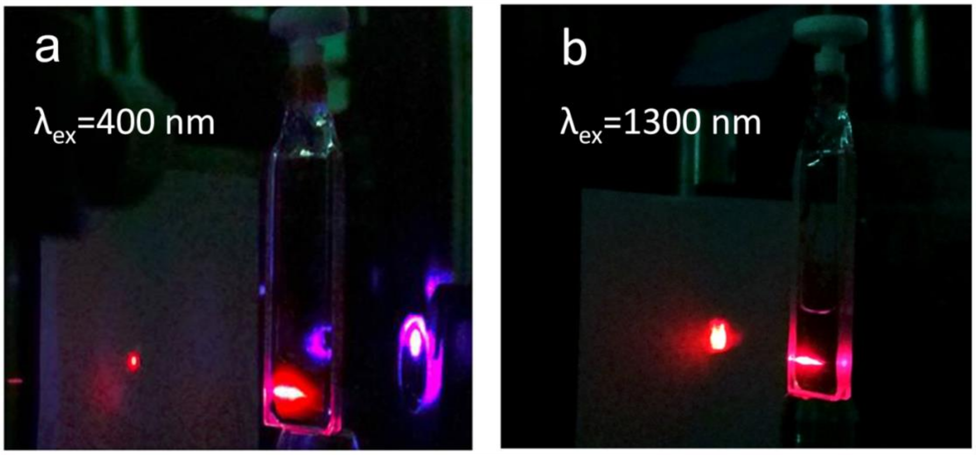

Supplementary Figure 1| One-photon and three-photon pumped lasing. Photographs of (**a**) one-photon pumped (λ_{exc} = 400 nm) lasing and (**b**) three-photon pumped (λ_{exc} = 1300 nm) lasing from NPLs solution in a cuvette. The smaller lasing beam spot on the screen under one-photon excitation is due to a much shallower penetration depth of 400 nm light.

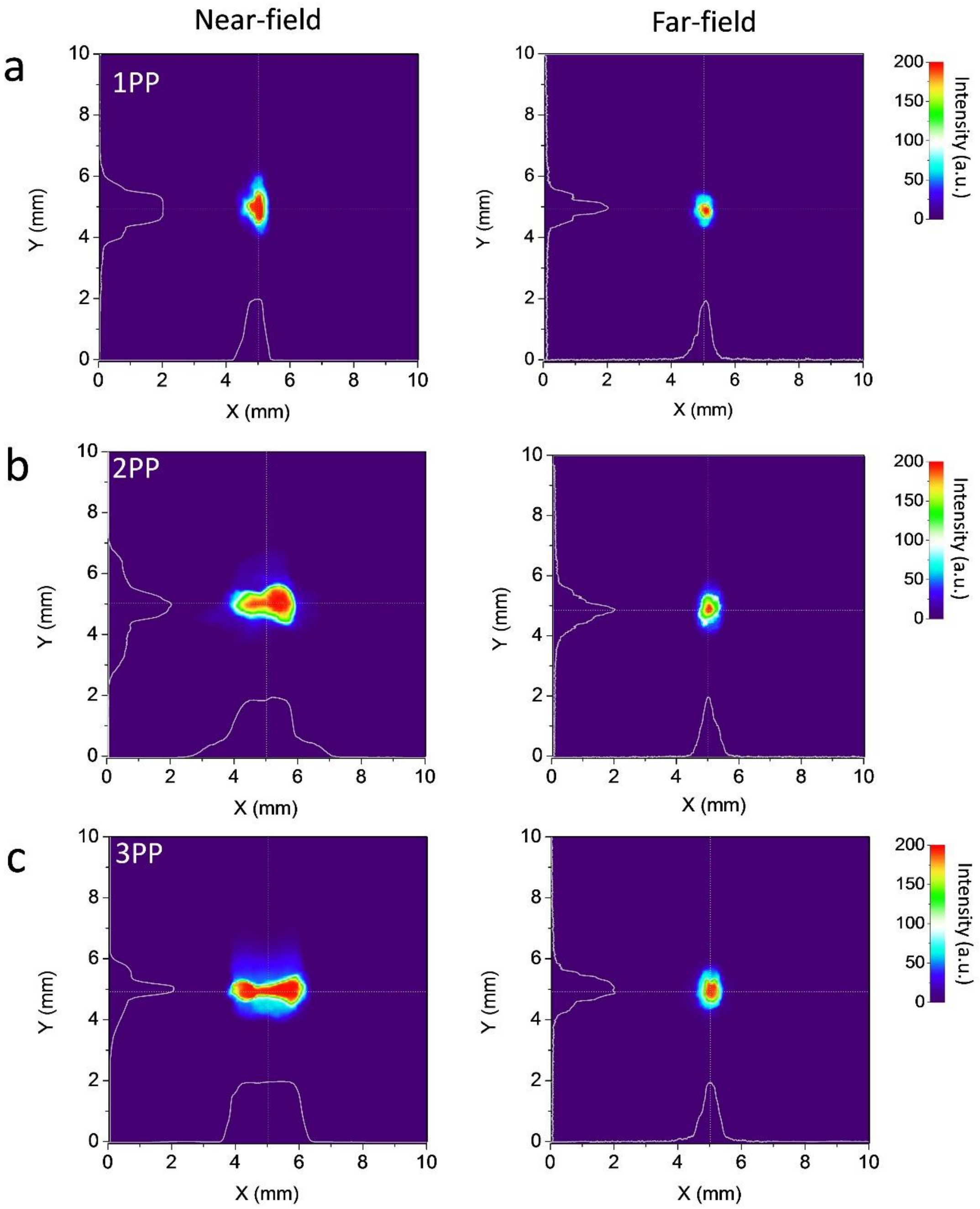

Supplementary Figure 2| Near-field and far-field intensity distributions. Near-field (left) and far-field (right) intensity distributions of the cuvette-based laser with the sample NPL390 in AP solution (with concentration of 21.6 μM) under (**a**) 1PP, (**b**) 2PP and (**c**) 3PP excitation. The gray curves at the bottom of the x- and y-axis represent the density profiles in the horizontal and vertical directions, respectively.

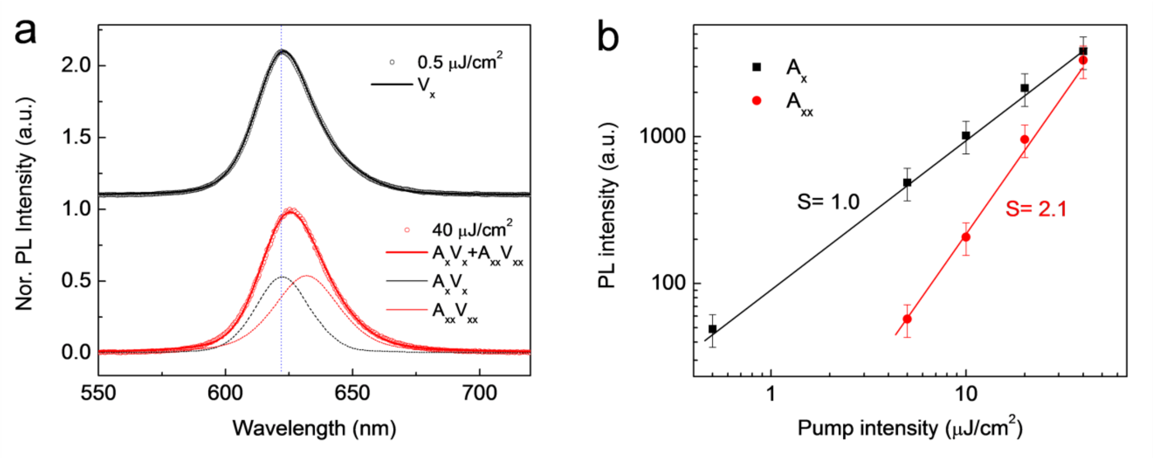

Supplementary Figure 3 | Biexciton binding energy determination. (**a**) Normalized static PL spectra of diluted sample NPL390 excited with 400 nm light under low (black circle dot) and high (red circle dot) pump fluence. The short dash lines are fittings. Vertical blue dot line is the guide for eyes. (**b**) Fitted amplitude A_{x} and A_{xx} as a function of pump intensity (S=slope). The error bars were determined by the uncertainty in the fit of the different PL intensities.

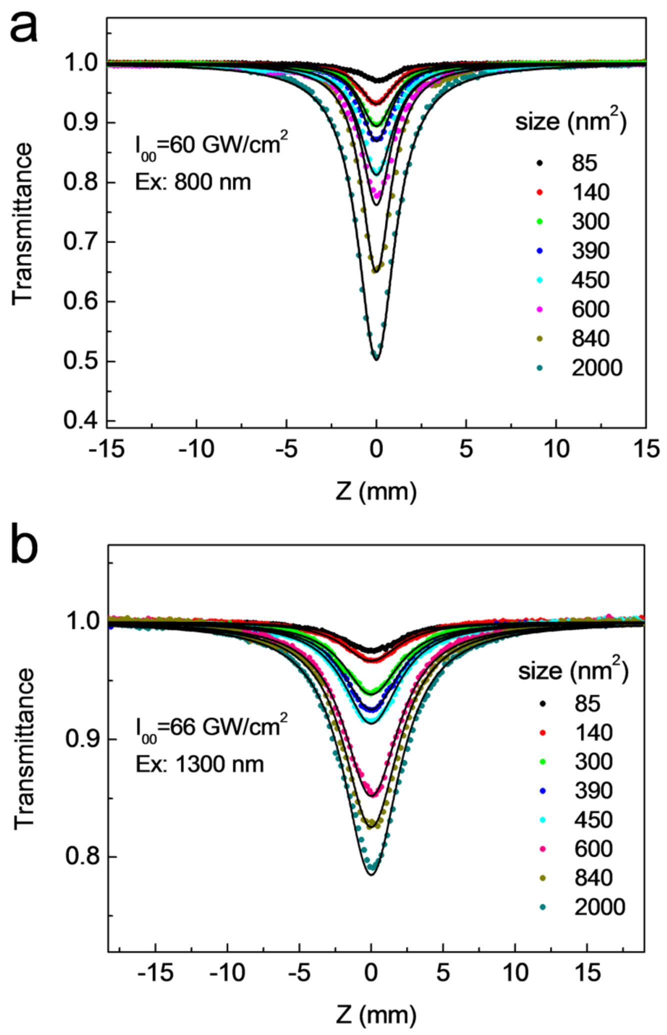

Supplementary Figure 4 | Z-scan measurements. Open-aperture Z-scan curves of NPLs with different lateral sizes in AP solution under: (**a**) two-photon excitation at 800 nm; (**b**) three-photon excitation at 1300 nm. Solid lines are fitting curves.

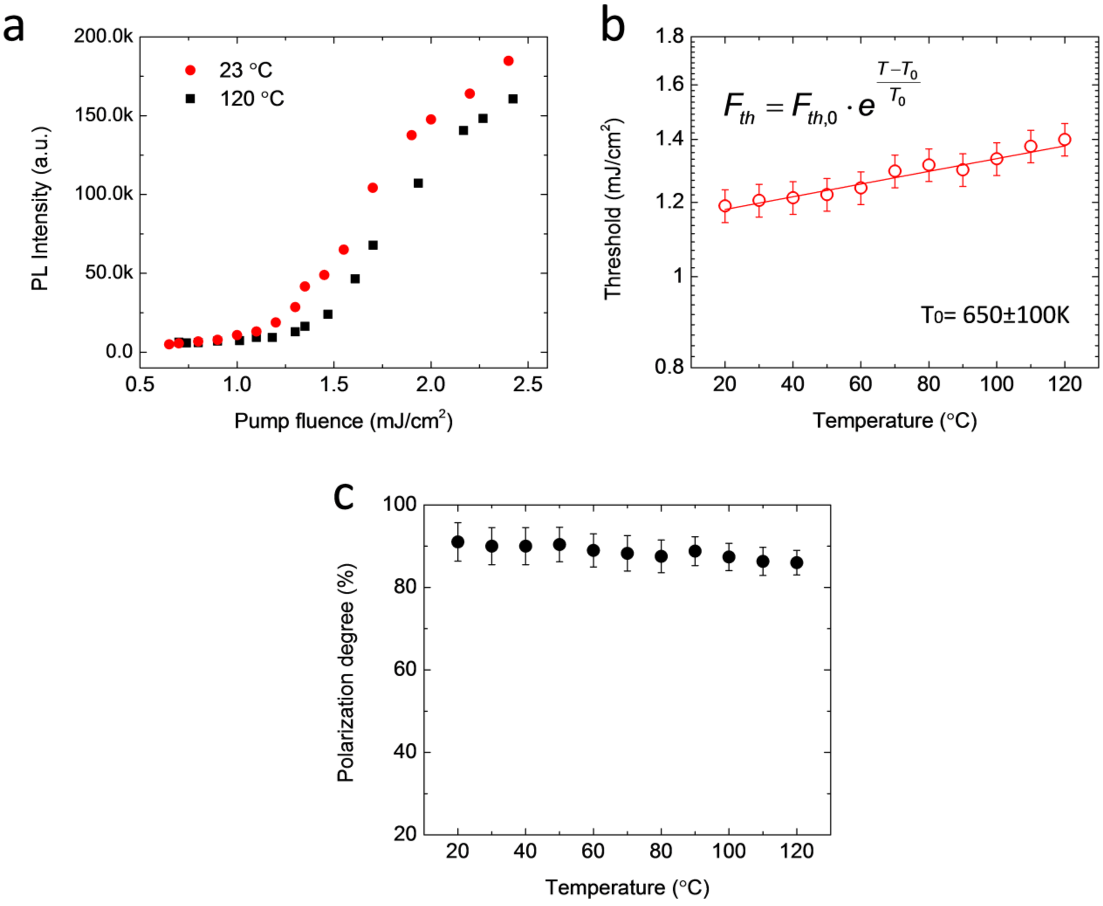

Supplementary Figure 5 | Thermal stability of 2PP lasing. Effect of temperature on the lasing threshold and polarization. (**a**) 2PP PL intensity of sample NPL390 in AP solution (with concentration of 21.6 μM) as a function of pump fluence at room-temperature (23 °C) and 120 °C. Temperature dependence of (**b**) threshold pump fluence (F_{th}) and (**c**) degree of polarization. The error bars were determined based on the standard deviation of 4 repeated acquisitions.

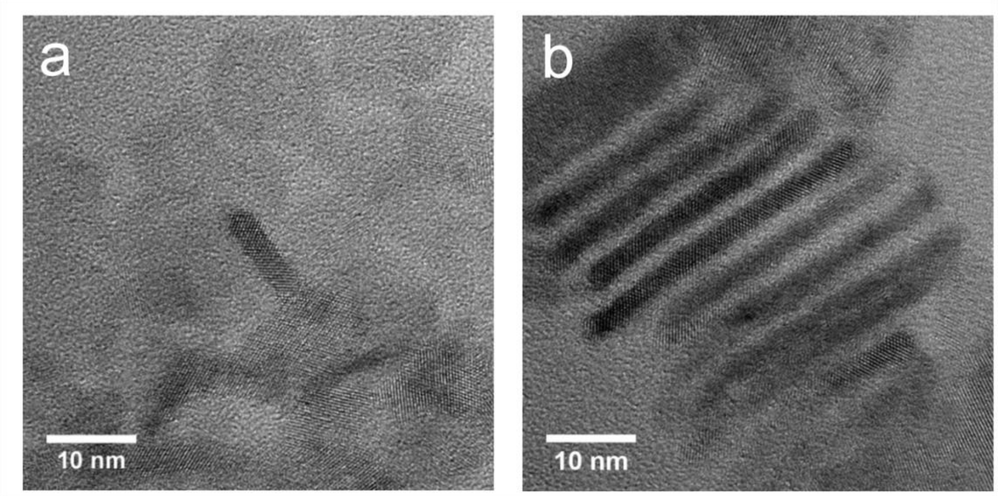

Supplementary Figure 6 | HRTEM of CdSe/CdS NPLs. High resolution TEM images of standing NPLs with average lateral size of (**a**) 85 and (**b**) 840 nm^2. From vertically standing NPLs, the thickness could be directly measured. A thickness value of ~2.75 nm was found for the different-sized NPLs.

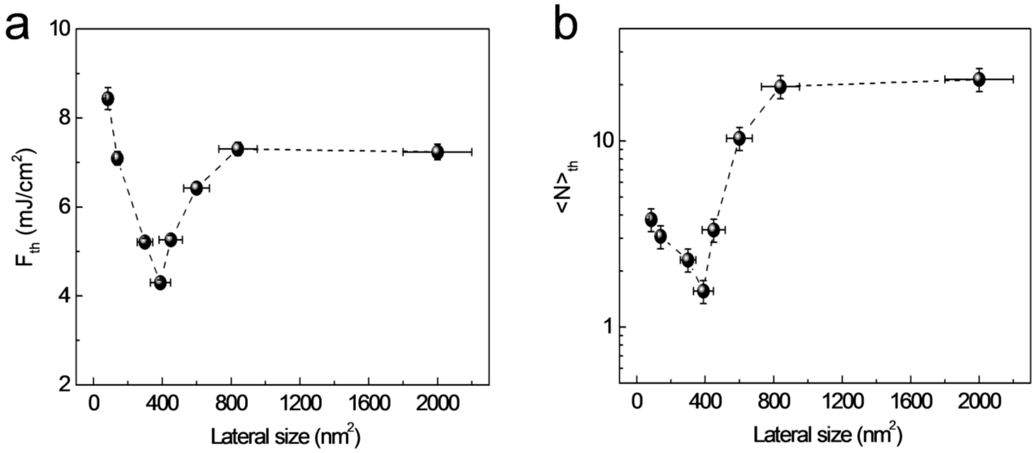

Supplementary Figure 7 | Threshold pump fluence and <N>$_{th}$ of three photon pumped lasing. (**a**) Threshold pump fluence F_{th} and (**b**) the average number of excitons per NPL, <N>$_{th}$, as a function of the lateral size of NPLs with concentration C_0. The samples were excited with 1300 nm light. The horizontal error bars were determined based on the lateral size distribution of the NPLs while the vertical error bars were determined based on the standard deviation of 4 repeated acquisitions.

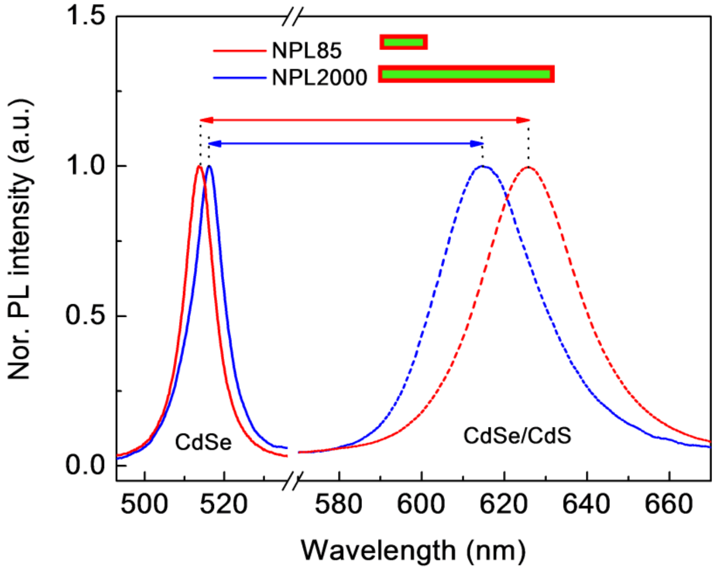

Supplementary Figure 8 | Effect of quantum confinement on PL of NPLs. Comparison of PL spectra of NPLs with average lateral sizes of 85 nm^2 (red) and 2000 nm^2 (blue) but with the same thickness. Solid lines refer to CdSe core NPLs, while dotted lines refer to CdSe/CdS core/shell NPLs. The cartoons illustrate the cross-section of the different sized CdSe/CdS NPLs, where the CdSe core is in green and the CdS shell is in red.

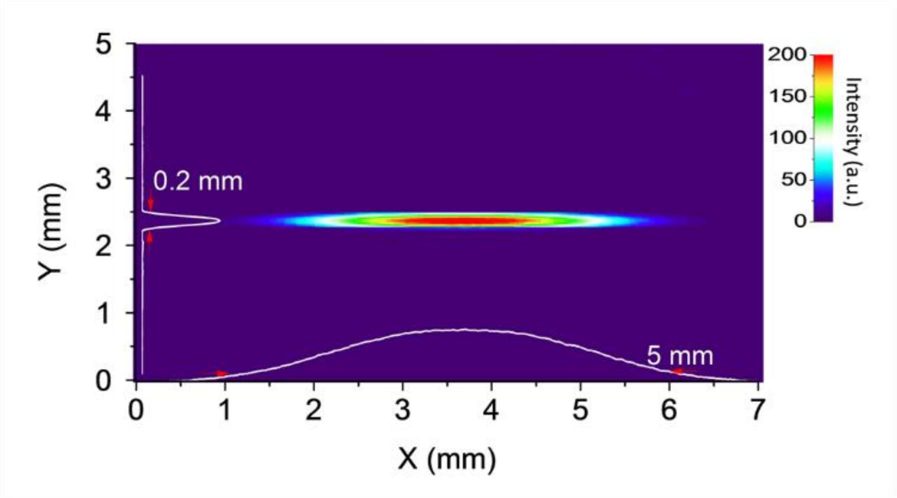

Supplementary Figure 9 | Pump beam intensity profile. Beam size (5 mm × 0.2 mm) is determined at the $1/e^2$ peak intensity.

Excitation wavelength	Near-field beam size (mm)		Far-field beam divergence angle (mrad)	
	horizontal	vertical	horizontal	vertical
400 nm (1PP)	0.7	1.1	2.2	2.5
800 nm (2PP)	2.0	1.0	2.8	4.0
1300 nm (3PP)	2.2	0.5	3.3	4.5

Supplementary Table 1 | Summary of measured near-field beam size measured at the half-maximum intensity, and the far-field divergence angle determined at the half-maximum intensity.

Supplementary Note 1 | Determination of CdSe/CdS NPL concentration

To ascertain the concentration of CdSe/CdS NPLs in solution, we first determined the concentration of CdSe NPLs before overcoating, which was calculated according to the equation[1]: $[NPL_{CdSe}]=2303A/(\sigma_{abs}N_A L)$, where A is the absorbance of the solution. σ_{abs} is the absorption cross-section, N_A is Avogadro's number, and L is the optical path length of the cuvette used in the absorbance measurement. It was previously found that at photon energies much larger than the band gap of semiconductor NCs, the effect of quantum confinement on molar extinction coefficient ε is negligible. Subsequently, it was suggested that ε at such wavelengths increases linearly with NC volume, in agreement with theoretical calculations and bulk values[1,2,3]. In our measurements, absorbances at a photon energy of 3.1 eV (which is well above the band gap of CdSe) were used to determine the σ_{abs} of CdSe NPLs. Using a previously reported value of σ_{abs} = 3.1×10^{-14} cm^2 at λ=400 nm for a 4 ML CdSe NPL with a lateral area of ~184 nm^2 as reference[3], the

σ_{abs} at λ=400 nm for 4 ML CdSe NPLs of different lateral sizes in our measurements were determined by scaling linearly the reference value of σ_{abs} with the ratio between the volume of the sample and that of the reference.

The concentration of CdSe/CdS NPLs were assumed to be approximately the same as the initial concentration of CdSe NPLs used. The loss of CdSe NPLs during overcoating with CdS was negligible due to the fact that no absorption or emission was observed from the supernatant during precipitation of the CdSe/CdS NPLs from their growth solution, thereby validating our assumption.

Supplementary Note 2 | Near-filed and far-field intensity distribution.

The near-field intensity distribution of lasing was obtained by imaging the emitting cuvette facet on the CCD camera using a f = 5 cm lens. To measure the far-field intensity distribution, the re-collimated output lasing beam after travelling a distance of about 2 meters was then focused onto the CCD camera placed at the focal plane position of a f = 150 cm lens. All laser emission intensities were reduced by a density filter before exposure to the CCD camera.

At the pump level of 1.5F_{th} for 2PP lasing, the measured near-field beam size at half-maximum intensity is ~2 mm in horizontal (x-axis) direction, and 1 mm in vertical (y-axis) direction. The far-field full divergence angles determined at half-maximum intensity are ~2.8 mrad in horizontal (x-axis) direction and ~4.0 mrad in vertical (y-axis) direction, respectively. The near-field beam size and far-filed divergence angle are larger under excitation with the longer pump laser wavelength, which is due to the increased

penetration depth (see Supplementary Fig. 2). The near-field beam size and far-field divergence angles of 1PP, 2PP and 3PP lasing at a pump level of ~1.5F_{th} are summarized in the Supplementary Table 1.

Supplementary Note 3 | Biexciton binding energy determination.

With increasing pump intensity, the PL spectra of a diluted solution of NPLs red-shifted (see Supplementary Figure 3). The PL spectrum at large pump intensities are fitted by the sum of two Pseudo-Voigt functions $A_x V_x + A_{xx} V_{xx}$, where A_X and A_{XX} represent the amplitude of the exciton (X) and biexciton (XX) emission respectively. The Pseudo-Voigt peak function is:

$$V = m_u \frac{2}{\pi} \frac{w}{4(\lambda - \lambda_0)^2 + w^2} + (1 - m_u) \frac{\sqrt{4 \ln 2}}{\sqrt{\pi} w} e^{-\frac{4 \ln 2}{w^2}(\lambda - \lambda_0)^2} \quad (1)$$

where the left and right terms are the Lorentzian and Gaussian profiles with relative contributions m_u and (1- m_u), and w is the line width. During the fitting, the variable λ_0 of V_x is fixed at the peak position of the PL spectrum obtained at very low pump intensity.

The amplitude of the fitted band on the lower energy side of the PL spectra was found to be quadratically dependent on the pump intensity ($A_{XX} \propto I^{2.1}$), which is indicative that the red band is due to a biexciton transition. The biexciton binding energy of about = 30 $\pm$0.5 meV is obtained from the energy difference between the two peak positions of V_x and V_{xx}.

Supplementary Note 4 | Z-scan measurements

According to the open aperture Z-scan theory for two-photon absorption (2PA) and three photon absorption (3PA), the normalized transmittance can be described respectively as[4,5]:

$$T_{2PA}(z) = \frac{1}{\pi^{1/2} q_0} \int_{-\infty}^{\infty} \ln[1 + q_0 \exp(-x^2)] dx \quad (2)$$

$$T_{3PA}(z) = \frac{1}{\pi^{1/2} q_0} \int_{-\infty}^{\infty} \ln\left\{[1 + p_0^2 \exp(-2x^2)]^{1/2} + p_0 \exp(-x^2)\right\} dx \quad (3)$$

where $q_0 = \alpha_2 I_0 L_{eff}$, $p_0 = (2\alpha_3 I_0^2 L'_{eff})^{1/2}$, $L_{eff} = [1 - \exp(-\alpha_0 L)] / \alpha_0$, $L'_{eff} = [1 - \exp(-2\alpha_0 L)] / 2\alpha_0$, $I_0 = I_{00} / (1 + z^2 / z_0^2)$, I_{00} is the on axis peak power and $z_0 = \pi \varpi_0^2 / \lambda$ is the Rayleigh range. α_0 , α_2 and α_3 are the linear, 2PA and 3PA coefficients, respectively.

Supplementary Fig. 4 shows the open aperture Z-scan curves of NPLs of different dimensions in a solution of AP. From the best fits of the data with equations (2) and (3), α_2 and α_3 can be extracted respectively. We then obtain the 2PA cross section (σ_2) and 3PA cross section (σ_3) by using the relations: $\sigma_2 = \alpha_2 \hbar \varpi / N$, $\sigma_3 = \alpha_3 (\hbar \varpi)^2 / N$. The calculated σ_2 and σ_3 are summarized in Table 1 of the main text.

Supplementary Note 5 | Determination of optical gain by pump-probe measurements

The single-pass gain g can be deduced by the following formulas based on Beer's law[6,7]:

$$I_{\mathrm{off}} = I_0 \mathrm{e}^{-\alpha_{\mathrm{int}} d} \tag{4}$$

$$I_{\mathrm{on}} - I_{\mathrm{pl}} = I_0 \mathrm{e}^{g d' - \alpha_{\mathrm{int}} d} \tag{5}$$

where I_0, I_{on}, I_{off} and I_{pl} are respectively the intensities of the incident beam, the transmitted probe beam with ("on") and without ("off") the pump beam, and the PL intensity generated by the pump beam. The wavelength of the probe beam was set to 634 nm (same as the NPL lasing peak). Here, d and d' are the sample optical pathlengths of the probe beam in the absence and presence of the pump beam, respectively. d corresponds to the optical pathlength of the cuvette for the pump probe measurement (= 200 μm) and d' was determined to be ~ 40 μm for our pump-probe measurement. α_{int} is the internal loss coefficient per unit length primarily due to free carrier absorption.

Supplementary Note 6 | Thermal stability of 2PP lasing.

To carry out temperature-dependent 2PP lasing measurements in solution, one side of the cuvette was attached to a copper sample holder of a cryostat using a silver adhesive that allowed for good heat transfer. The 2PP lasing threshold was found to be relatively stable with increasing temperature from 23 °C (296 K) to 120 °C (393 K). As shown in Supplementary Figs. 5a and 5b, the threshold only increases slightly from ~1.2 to 1.4 mJ cm^{-2}, indicating a nearly temperature-independent lasing threshold within the

range of temperatures used. The dependence of F_{th} on temperature is fitted by the formula of [8]:

$$F_{\mathrm{th}} = F_{\mathrm{th},0} \cdot e^{\frac{T-T_0}{T_0}} , \qquad (6)$$

where the fitted value T_0 represents a characteristic temperature of the laser. A large value of T_0 is usually indicative of a nearly temperature-independent threshold. Here, T_0 is obtained to be 650 ± 100 K. which is larger than those of quantum well film-based lasers (~100 K) and close to the previous reported ones for ASE in close-packed thin films of colloidal CdSe/CdS dots and nanorods (~350-950 K)[8].

The effect of temperature on the degree of polarization (which is given by $p = \frac{I\| - I\perp}{I\| + I\perp}$) of 2PP lasing was also investigated (See Supplementary Fig. 5c). The high degree of polarization of MPP lasing is supported by the anisotropic optical properties of NPLs[9] and the high viscosity of the AP solvent. Due to the high viscosity of the AP solvent, the reorientation time for the NPLs should be much longer compared to the pump pulse duration and lasing lifetime of the NPL, thus reorientation of the NPL during pumping and emission could be neglected. Since the degree of polarization is dependent on viscosity, the nearly temperature-independent polarization is likely due to minimal changes in the viscosity of the AP solvent from 23°C to 120°C.

Supplementary Methods

Small CdSe nanoplatelets (NPLs) with lateral areas (< 200 nm^2) were synthesized based on a previously reported method[10]. Briefly, in a three-neck round bottom flask (RBF), 170 mg of cadmium myristate, 12 mg of selenium powder and 15 ml of 1-octadecene (ODE) were degassed under vacuum at 80 °C for 1 hr. The mixture was subsequently heated up to 240 °C and 80 mg of cadmium acetate was introduced into the RBF at around 195 °C when the solution turned orange. The reaction mixture was kept at 240 °C for 8 min and after the reaction had cooled down 1 ml of oleic acid was injected to the solution. The 4 monolayer (ML) NPLs were processed via two cycles of dispersion and precipitation with hexane and ethanol respectively. CdSe NPLs with larger lateral areas (> 300 nm^2) were synthesized using a seeded-extension method[11]. Firstly, processed CdSe NPL seeds with lateral dimensions of ~30 nm × 7 nm were prepared via the same procedure as that for small NPLs and dispersed in 4 ml of hexane. 3 ml of 2.4 μM seed NPL solution was introduced into a RBF together with 8 ml of ODE. The mixture was then degassed at room temperature for 20 min and further degassed at 80 °C for 30 min. Then temperature was subsequently increased to 240 °C and a mixture of cadmium acetate dissolved in ODE (0.1 mM) and selenium powder dissolved in ODE (0.12 mM) was injected to the RBF at a rate of 30 ml h^{-1}. By this approach, NPLs with different lateral sizes larger than 300 nm^2 could be obtained by injecting various amount of Cd-ODE and Se-ODE. For example, NPLs with a lateral area of 300 nm^2 were synthesized using 2 ml of Cd-ODE solution and 2 ml of Se-ODE. By adjusting the relative amounts of Cd to Se solution added (e.g., 1:2), larger NPLs of the same thickness could be synthesized.

Core-shell CdSe/CdS NPLs were synthesized by overcoating several monolayers (MLs) of CdS as a shell on CdSe NPLs via an atomic layer deposition approach[12] with slight modifications. Firstly, CdSe NPLs as prepared above was dissolved in 4 ml of hexane and then the S layer was grown by introducing 4 ml of 0.06 M ammonium sulfide in N-methylformamide (NMF) and 2 ml of NMF into the mixture. Excess free S^{2-} was removed by adding toluene, centrifuging the mixture and removing the supernatant. The NPLs were then dispersed in 3 ml of NMF. To grow the Cd layer, 1.5 ml of cadmium acetate solution (0.25 M in NMF) was added. After stirring for 5 min, the NPLs were precipitated with toluene and acetonitrile. Finally, 1 ML of CdS shell was completely grown on the CdSe NPLs. By repeating the above-mentioned overcoating steps, CdSe NPLs with 2 ML thick CdS shells were obtained. The as-synthesized CdSe/CdS NPLs were processed from the growth solution via the same procedure as for CdSe NPLs, and the residual solvent was removed under vacuum. The NPLs were then dispersed in neat 5-amino-1-pentanol (AP) (Alfa Aesar, 97%) and used for lasing measurements.

Supplementary References

[1] Leatherdale, C. A., Woo, W. K., Mikulec, F. V. and Bawendi, M. G. On the absorption cross section of CdSe nanocrystal quantum dots. *J. Phys. Chem. B* **106,** 7619 (2002).

[2] Moreels, I. *et al.* Size-dependent optical properties of colloidal PbS quantum dots. *ACS nano* **3,** 3023 (2009).

[3] Shaviv, E., Salant, A. and Banin, U. Size dependence of molar absorption coefficients of CdSe semiconductor quantum rods. *ChemPhysChem*, **10,** 1028 (2009).

[4] Xing, G. *et al*. Enhanced tunability of the multiphoton absorption cross-section in seeded CdSe/CdS nanorod heterostructures. *Appl. Phys. Lett*. **97,** 061112 (2010).

[5] Xing, G. *et al*. Three-Photon Absorption in Seeded CdSe/CdS Nanorod Heterostructures. *J. Phys. Chem. C* **115,** 17711 (2011).

[6] Pavesi, L., Dal Negro, L., Mazzoleni, C., Franzo, G. and Priolo, F. Optical gain in silicon nanocrystals. *Nature*, **408,** 440 (2000).

[7] Dal Negro. L. *et al*. Light amplification in silicon nanocrystals by pump and probe transmission measurements. *J. Appl. Phys.* **96,** 5747 (2004).

[8] Moreels, I. *et al*. Nearly Temperature-Independent Threshold for Amplified Spontaneous Emission in Colloidal CdSe/CdS Quantum Dot-in-Rods. *Adv. Mat*. **24,** OP231 (2012).

[9] Abecassis, B. *et al*. Self-Assembly of CdSe Nanoplatelets into Giant Micrometer-Scale Needles Emitting Polarized Light. *Nano Lett*. **14,** 710 (2014).

[10] Tessier, M. D. *et al*. Efficient Exciton Concentrators Built from Colloidal Core/Crown CdSe/CdS Semiconductor Nanoplatelets. *Nano Lett*. **14,** 207 (2013).

[11] Bouet, C. *et al*. Two-dimensional growth of CdSe nanocrystals, from nanoplatelets to nanosheets. *Chem. Mat*. **25,** 639 (2013).

[12] She, C. *et al*. Low-threshold stimulated emission using colloidal quantum wells. *Nano Lett*. **14,** 2772 (2014).
